# Perceived social support and deviant behavior of new street corner youth on campus: a moderated mediation model

**DOI:** 10.3389/fpsyg.2024.1430482

**Published:** 2024-11-22

**Authors:** Hong Chi, Linlin Fan, Weijie Meng

**Affiliations:** ^1^College of Education, Ludong University, Yantai, China; ^2^School of Resources and Environmental Engineering, Ludong University, Yantai, China; ^3^School of Marxism, Shandong Technology and Business University, Yantai, China

**Keywords:** perceived social support, interpersonal needs, meaning in life, deviant behavior, new street corner youth on campus

## Abstract

**Introduction:**

The current study aimed to examine the moderated mediation between perceived social support, the deviant behavior of new street corner youth on campus (NSCYC), interpersonal needs (as a mediator), and meaning in life (as a moderator).

**Methods:**

A total of 596 new street corner youth on campus were investigated using the Deviant Behavior Questionnaire, Perceived Social Support Scale, Interpersonal Needs Questionnaire, and Meaning in Life Questionnaire.

**Results:**

The findings showed that: (1) perceived social support significantly and negatively predicted deviant behavior; (2) interpersonal needs mediated the association between perceived social support and deviant behavior; and (3) a moderated mediating role of perceived social support influencing deviant behavior was established, with meaning in life regulating both the direct and the first half paths of the model.

**Discussion:**

These findings offer important insights into the factors influencing the deviant behavior of NSCYC. They highlight the role of perceived social support, interpersonal needs, and meaning in life, and suggesting intervention points to mitigate deviant behaviors of NSCYC.

## Introduction

Street corner youth are a marginalized group of adolescents with notable sub-criminal characteristics, which render them distanced from community building and mainstream society. They lack stable and reliable sources of income, have a stable internal organizational structure, and are considered socially harmful (Huang, 2007). Previous research has suggested that some students on campus communicate with street corner youth, referring to these students as new street corner youth on campus (NSCYC) (Fan and Meng, 2022). Relevant studies have shown that students who associate with street corner youth exhibit more deviant behavior. For instance, 46% of these students reported refusing to attend school, while 47% showed patterns of absenteeism and being related to criminal behaviors (Olley, 2006). The most prominent manifestation of NSCYC is their behavior, which is fundamentally transgressive and deviant (Karabanow and Clement, 2004). Deviant behavior refers to actions that violate school discipline, deviate from social and moral norms, and harm the healthy development of individuals and society. These behaviors are considered abnormal and maladaptive (Li Y. J. et al., 2015). Adolescents’ deviant behavior not only disrupts the normal socialization process and social stability but also endangers the safety of others (Dullas et al., 2021). The deviant behavior of new street corner youth on campus is characterized by aversion and truancy, emotional sensitivity, and close interaction with street corner youth, which often lead to fights and bullying on campus and even result in delinquency (Fu and Wen, 2004). To prevent NSCYC from consuming social resources and causing serious social problems in the future, the deviant behavior of NSCYC should be detected and corrected as early as possible. Accordingly, there is a necessity to investigate the factors and mechanisms influencing this behavior, which is important for preventing and intervening in deviant behavior among NSCYC.

### Perceived social support and deviant behavior

Perceived social support refers to the subjective experience of feeling respected, supported, and well-understood within society (Norris and Kaniasty, 1996). Social support from parents, teachers, and peers facilitates the positive development of adolescents’ future orientation and serves as a significant protective factor for their healthy psychological development (Miloseva et al., 2017; Chen et al., 2024). A longitudinal survey suggested that greater social support is associated with less aggressive behavior in adolescents 1 year later (Wright and Wachs, 2019). In addition, it has been found that adequate social support helps adolescents acquire more coping strategies (Reife et al., 2020), which helps them prevent and improve negative behaviors, as well as reduce negative emotions and their adverse effects (Ma Y. et al., 2022). NSCYC do not receive the warmth of their family, experience poor teacher–student relationships at school, and face exclusion from their classmates, resulting in limited social support (Fu, 2003).

### Interpersonal needs as the mediator

Interpersonal needs refer to the need for interpersonal interaction that emerges during an individual’s social adjustment process (Joiner and VanOrden, 2008). When interpersonal needs are not met, it can lead to a frustrated sense of belonging and feelings of fatigue, triggering maladaptive behaviors (Jin et al., 2016). It has been found that there are several causes of interpersonal need deficits, such as parent–child conflict, poor peer relationships, negative social events, childhood abuse, and emotional neglect (Carcedo et al., 2008). When adolescents perceive less support from parents, teachers, and peers, their relational needs are less likely to be met, which negatively affects their interpersonal needs, leading to an increased sense of lack in these areas (Liu, 2021). The more serious the lack of interpersonal needs, the more likely the individuals are to engage in deviant behavior (Yang, 2003). Relevant empirical studies have shown that a lack of interpersonal needs serves as a precursor and an effective predictor of transgressive behavior (VanOrden et al., 2012). According to the Generalized Stress Theory, individuals exhibit maladaptive, deviant, or criminal behaviors mainly to relieve the tension generated by a lack of interpersonal needs. This behavior helps individuals alleviate stress, reduce or avoid negative stimuli, and maintain a balanced cognitive and emotional state (Agnew, 1985). In addition, a study found that interpersonal needs play a mediating role between social support and low self-esteem and that adequate social support decreases individuals’ negative emotional experiences, alleviates the lack of belonging, and reduces low self-esteem by fulfilling interpersonal needs (Song et al., 2022). Therefore, interpersonal needs may play a mediating role between the perception of social support and deviant behavior.

### Meaning in life as the moderator

Meaning in life refers to an individual’s recognition of the significance of life, as well as the understanding and pursuit of the purpose of existence, aimed at realizing the value of their own life (Steger et al., 2006). Individuals with a strong sense of meaning in life are more likely to tap into their internal potential, make clear and rational plans for their life goals and directions, and enhance their sense of life’s value (Yang, 2022). Previous studies have found that individuals with a stronger sense of meaning in life maintain a positive and optimistic attitude toward their lives, have a higher level of self-identification, and feel supported and valued by their parents, teachers, and peers. These factors contribute to the formation of harmonious interpersonal relationships, enhance the sense of belonging, and positively impact the satisfaction of interpersonal needs (Zhang and Peng, 2022). When perceived social support is low, individuals with a stronger sense of meaning in life have higher subjective well-being, can plan their lives rationally, and dedicate themselves to pursuing their goals, resulting in a reduced sense of lacking interpersonal needs (Chen et al., 2020). In contrast, individuals with a lower sense of meaning in life are more likely to experience psychological pain. When they perceive lower levels of social support, they tend to view themselves negatively and lack hope for the future (Nie and Gan, 2017). When individuals’ needs for belonging are not met, it leads to negative imbalances and a more pronounced lack of interpersonal needs (Cao et al., 2023).

At the same time, the present study suggested that meaning in life may play a moderating role in the relationship between perceived social support and deviant behavior. Individuals with a stronger sense of meaning in life gain strong internal motivation to focus on creating value in life and abandoning deviant behavior (Chen and Wu, 2014). When experiencing lower social support, individuals with a stronger sense of meaning in life still maintain hope for the future. They seek interpersonal interactions through appropriate and reasonable means, exercise effective self-control, and engage in less deviant behavior (Cheng et al., 2019). It was found that the absence of meaning in life correlates closely with various addictive behaviors (Melton and Schulenberg, 2007; Zhang et al., 2019). When individuals perceive less social support, their sense of social connection and self-control weakens. This can lead them to adopt unhealthy methods to relieve the tension caused by a lack of interpersonal needs, resulting in an increase in deviant behavior (Zhao et al., 2020).

Therefore, we propose the following hypotheses:

*Hypothesis 1*. Perceived social support is negatively associated with deviant behavior.

*Hypothesis 2*. Interpersonal needs play a mediating role between perceived social support and deviant behavior.

*Hypothesis 3*. Meaning in life moderates the relationship between perceived social support and interpersonal needs.

*Hypothesis 4*. Meaning in life moderates the relationship between perceived social support and deviant behavior.

**Figure 1:**
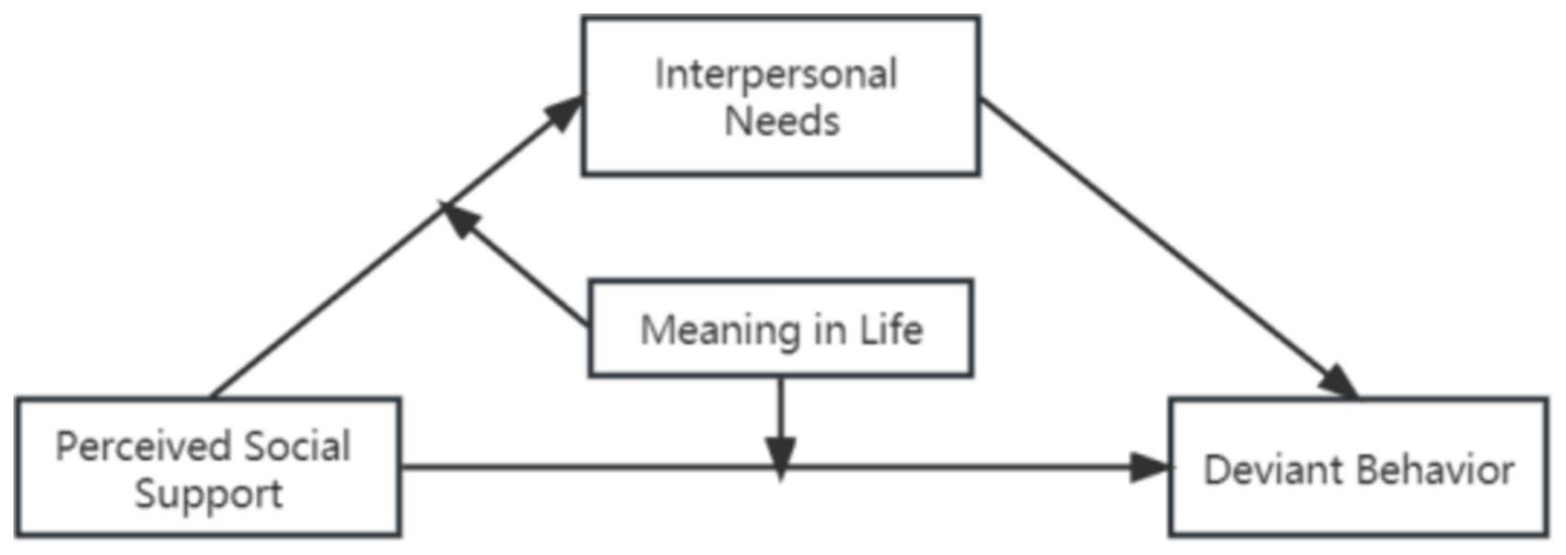
Theoretical model of the current research.

In summary, the present research has constructed a moderated mediation model to explore the effects of perceived social support on deviant behavior among NSCYC (see Figure 1) and to examine the mediating role of interpersonal needs and the moderating role of meaning in life in the relationship between perceived social support and deviant behavior.

## Methods

### Participants and procedure

In this study, questionnaires were used to survey NSCYC from 16 schools in China. For data collection, self-reported data were used. The experimenter purposefully selected participants who had the characteristics of NSCYC for the survey. Our criteria for selecting NSCYC included students who interacted with street corner youth, such as being friends with them and attending parties together. A total of 600 paper questionnaires were distributed, and 596 valid questionnaires were returned, resulting in a valid return rate of 99.33%. The mean age of the participants was 18.83 years (*SD* = 3.15). A total of 341 (57.2%) participants were men and 255 (42.8%) were women.

We obtained ethical approval from the institutional review board of the investigator’s university, and informed consent was obtained from the parents. The researcher read the survey instructions to the participants and requested them to carefully answer the paper questionnaires. The data were collected anonymously. After completing the questionnaires, each participant received a gift as compensation.

### Measures

#### Deviant behavior

Deviant behavior was measured using the student deviance section of the China Education Panel Survey (CEPS), which has been widely used in China and demonstrates high reliability (Ke, 2022). It is a 10-item questionnaire that functions as a one-dimensional scale, with items such as “bullying weak classmates.” The participants rated all items on a 5-point scale (1 = never to 5 = always), with higher scores indicating more deviant behavior. The internal consistency was 0.85.

#### Perceived social support

Perceived social support was measured using the Perceived Social Support Scale revised by Yan and Zheng (2006). It is a 12-item questionnaire consisting of three dimensions—family support, friend support, and other support. The participants rated all items on a 7-point scale (1 = totally disagree to 7 = totally agree), with items such as “My friends can share my happiness and sadness.” Higher scores were associated with a greater level of perceived social support. The internal consistency was 0.97.

#### Interpersonal needs

Interpersonal needs were measured using the Interpersonal Needs Questionnaire developed by Li X. M. et al. (2015). This 15-item questionnaire consists of two dimensions—lack of belonging and a sense of tiredness. The participants rated all items on a 7-point scale (1 = totally disagree to 7 = totally agree), with items such as, “I am close to others.” Higher scores indicated that fewer interpersonal needs were met. The internal consistency was 0.90.

#### Meaning in life

Meaning in life was assessed using the Meaning in Life Questionnaire developed by Steger et al. (2006) and revised by Wang (2013). This 10-item questionnaire consists of two dimensions—life meaning experience and life meaning seeking. The participants rated the items on a 7-point scale (1 = totally disagree to 7 = totally agree), with items such as, “I’m looking for a purpose or mission in my life.” Higher scores were associated with greater meaning in life. The internal consistency was 0.90.

In this study, SPSS 26.0 was used for data analysis, and descriptive statistical analysis of the data and a common method deviation test were carried out. The mediation model was verified using the PROCESS Macro Model 4, and the moderated mediation model was verified using the PROCESS Macro Model 59.

## Results

### Preliminary analysis

A correlation analysis of the variables showed that perceived social support was negatively related to both interpersonal needs and deviant behavior, while positively correlated with meaning in life. Interpersonal needs were positively related to deviant behavior, while negatively related to meaning in life. Meaning in life was negatively related to deviant behavior (Table 1).

**Table 1:** Descriptive statistics and correlations between variables.

	*M*	*SD*	Skewness	Kurtosis	Gender	Age	1	2	3	4
Gender	–	–			1					
Age	18.83	3.15			−0.04	1				
Perceived social support	64.54	15.37	−1.128	1.649	0.14^**^	−0.07	1			
Interpersonal needs	37.00	16.56	0.894	0.38	−0.17^**^	0.08	−0.58^**^	1		
Meaning in life	49.06	11.79	−0.404	0.342	0.11^**^	−0.12^**^	0.59^**^	−0.49^**^	1	
Deviant behavior	15.14	5.37	1.899	5.994	−0.09^*^	0.19^**^	−0.13^**^	0.24^**^	−0.10^*^	1

*N* = 596, ^⁎^*p* < 0.05; ^**^p < 0.01; ^***^p < 0.001, same below.

### Testing for mediating role

Model 4 of the SPSS Macro PROCESS was used to centralize the variables included in the model, and non-parametric bootstrapping with 5,000 times repeated sampling was employed to test the mediating effect between perceived social support and the deviant behavior of the newly met young people on campus. Gender and age were included in the regression equation as control variables. Our findings indicated that perceived social support had a significant negative predictive effect on deviant behavior (*β* = −0.11, *p* < 0.01) and interpersonal needs (*β* = −0.56, *p* < 0.01). When interpersonal needs were included in the regression equation, they had a significant positive predictive effect on deviant behavior (Table 2). The 95% bootstrap confidence intervals for the mediating role of interpersonal needs were non-zero, suggesting a significant mediating role of interpersonal needs in the relationship between perceived social support and deviant behavior (ab = −0.13, SE = 0.04, 95%CI = [−0.20, −0.06]).

**Table 2:** Results of the mediation model.

Predictors	Deviant behavior		Interpersonal needs		Deviant behavior	
	*β*	*t*	*β*	*t*	*β*	*t*
Gender	−0.07	−1.74	−0.09	−2.55^*^	−0.05	−1.27
Age	0.18	4.53^***^	0.03	1.02	0.17	4.40^***^
Perceived social support	−0.11	−2.61^**^	−0.56	−16.49^***^	0.02	0.43
Interpersonal needs					0.23	4.68^***^
*R* ^2^	0.06		0.34		0.09	
F	11.47^***^		100.51^***^		14.39^***^	

### Testing for moderated mediation

Using the moderated mediation model of the SPSS PROCESS Macro, the moderating role of meaning in life was examined while controlling for gender and age. The findings were as follows (Table 3): the interaction term of perceived social support and meaning in life was a significant negative predictor of interpersonal needs (*β* = −0.10, *t* = −4.39, *p* < 0.001), and the interaction term between perceived social support and meaning in life significantly and negatively predicted deviant behavior (*β* = −0.11, *t* = −3.29, *p* < 0.05). Thus, meaning in life moderated the direct and the first half paths of interpersonal needs mediating perceived social support and deviant behavior.

**Table 3:** Moderated mediation analysis.

Predictors	Interpersonal needs		Deviant behavior	
	*β*	*t*	*β*	*t*
Gender	−0.08	−2.33^*^	−0.05	−1.20
Age	0.02	0.60	0.18	4.53^***^
Perceived social support	−0.49	−11.59^***^	−0.07	−1.31
Meaning in life	−0.23	−5.75^***^	0.01	0.20
Perceived social support × meaning in life	−0.10	−4.39^***^	−0.11	−3.29^**^
Interpersonal needs			0.20	4.00^***^
Interpersonal needs × meaning in life			−0.01	−0.12
*R* ^2^	0.39		0.11	
*F*	74.64^***^		10.56^***^	

**Figure 2:**
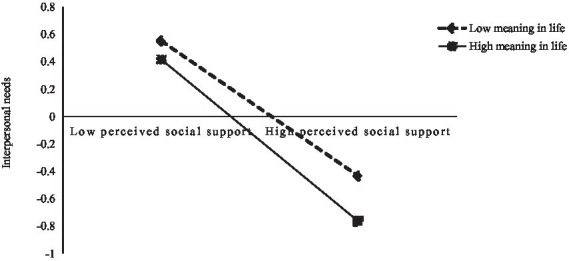
Moderating role of meaning in life between perceived social support and interpersonal needs.

**Figure 3:**
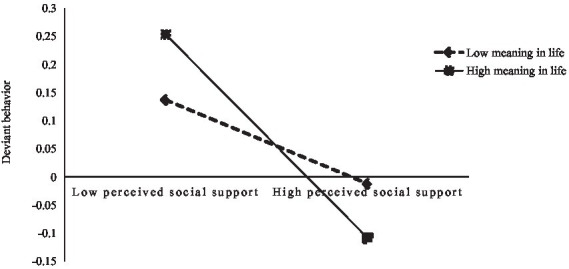
Moderating role of meaning in life between perceived social support and deviant behavior.

Simple slope tests were performed by taking the values of meaning in life plus and minus 1 standard deviation (Ma Z. L. et al., 2022; Su et al., 2022) (see Figures 2, 3). The results indicated that perceived social support was a significant negative predictor of interpersonal needs at high levels of meaning in life (simple slope = −0.60, *t* = −10.95, *p* < 0.001) and that a stronger predictive effect of perceived social support on interpersonal needs was significant at high levels of meaning in life (simple slope = −0.40, *t* = −9.60, *p* < 0.001), indicating that higher levels of meaning in life were associated with weaker effects of perceived social support on interpersonal needs. Meanwhile, perceived social support negatively predicted deviant behavior at high levels of meaning in life (simple slope = −0.18, *t* = −2.43, *p* < 0.05). Perceived social support had no significant predictive role on the deviant behavior of the NSCYC with a low sense of meaning in life (simple slope = 0.03, *t* = 0.58, *p* > 0.05).

## Discussion

### Relationship between perceived social support and deviant behavior

The present study found that perceived social support was significantly and negatively related to the deviant behavior of the NSCYC, indicating that the lower the level of perceived social support, the more deviant the behavior. This finding supports Hypothesis 1 and is consistent with the results of previous studies (Ma Y. et al., 2022; Mukhtar and Mahmood, 2018). Previous research has found that social support from parents, teachers, and peers is an important protective factor for adolescents dealing with cyberbullying and can be effective in improving their psychological well-being (Kowalski et al., 2014). Adolescents with higher levels of perceived social support exhibit less aggressive behavior (Rothon et al., 2011). The lack of warmth in the home environment and poor teacher–student relationships among NSCYC, along with social prejudice and discrimination, have weakened societal control and connection, leading to an increase in deviant behavior (Fu, 2003).

### Interpersonal needs as the mediator

It was found that interpersonal needs mediated the relationship between perceived social support and the deviant behavior of the NSCYC, indicating that a decrease in perceived social support can affect the deviant behavior of NSCYC by creating a lack of fulfillment of interpersonal needs. This finding supports hypothesis 2 and is consistent with previous results (Poindexter et al., 2015). This is because NSCYC perceive less social support, desire attention and support from parents, teachers, and peers, experience an unsatisfied sense of belonging, and face a greater lack of fulfillment of interpersonal needs (Fu, 2003). Previous research has suggested that unmet interpersonal needs have a positive predictive effect on adolescents’ deviant behavior, indicating that higher levels of interpersonal need deficits are associated with increased deviant behavior among adolescents (Jiang, 2013; VanOrden et al., 2012). Although the mediation analysis revealed the influence of social support and interpersonal needs on deviant behavior, the R^2^ value was relatively low. This is likely because the deviant behavior of NSCYC is not entirely dependent on these two factors; it is also influenced by other factors. Consequently, the predictors can explain only a small portion of the variance.

Thus, it is likely that NSCYC engage in deviant behavior as a way to relieve the tension caused by unmet interpersonal needs, seeking to gain the care and attention of parents, teachers, and classmates, enhance their sense of belonging, and fulfill their interpersonal needs.

### Moderation role of meaning in life

The present study found that meaning in life moderated the first half path of interpersonal needs mediating perceived social support and deviant behavior, a finding that supports hypothesis 3. This suggests that meaning in life reduces the effect of perceived social support on interpersonal needs. Individuals with a stronger sense of meaning in life can reduce internal psychological stress by pursuing life goals when faced with stressful life events (Lin et al., 2021). This means that new street corner youth on campus with a stronger sense of meaning in life can plan their lives rationally when they perceive a low level of social support, devote themselves to the pursuit of their goals, experience higher subjective well-being, and have relatively low levels of lack of unmet interpersonal needs (Zhang et al., 2021). In contrast, new street corner youth on campus with a low sense of meaning in life experience greater psychological pain when they perceive lower levels of social support, resulting in a significant deficiency in their sense of belonging and interpersonal needs (Nie and Gan, 2017). Thus, meaning in life reduces the impact of perceived social support on the interpersonal needs of NSCYC.

The present study found that meaning in life moderated the direct path of perceived social support and the deviant behavior of NSCYC, a finding that supports hypothesis 4. This suggests that meaning in life reduces the effect of perceived social support on deviant behavior. Specifically, NSCYC who have a stronger sense of meaning in life exhibit less deviant behavior when they perceive low levels of social support, whereas new corner youth on campus with a low sense of meaning in life exhibit more deviant behavior when they perceive low levels of social support. Previous research has shown that meaning in life can provide individuals with value for life and can effectively regulate their behavior and negative emotions (Lin et al., 2022). New street corner youth on campus with a stronger sense of meaning in life can effectively manage life events when they perceive low levels of social support, seek interpersonal interaction through appropriate and reasonable means, and exhibit less deviant behavior. In contrast, when new street corner youth on campus with a low sense of meaning in life perceive low social support, a lack of appropriate cognitive factors leads them to adopt wrong ways for relieving the tension caused by unmet interpersonal needs, resulting in an increase in deviant behavior (Zhao et al., 2020). Thus, meaning in life reduces the effect of perceived social support on the deviant behavior of NSCYC.

### Implications and limitations

To summarize, the current research examined the effects of perceived social support, interpersonal needs, and meaning in life on the deviant behavior of new street corner youth on campus. The findings from our study have important implications for preventing deviant behavior. First, we should enhance social support for NSCYC. Social support from family, school, and peers is important for the healthy development of these youth. Second, we need to focus on the interpersonal needs of NSCYC. Parents should pay attention to the frequency and methods of communication with these youth and actively engage in their growth process. Children require not only material support but also emotional support. Parents should also communicate with school teachers, learn about the situation of NSCYC in a timely manner, and discuss educational strategies with teachers.

The NSCYC often have unmet interpersonal needs and seek the companionship of street corner youth to gain a sense of belonging and identity. Finally, we should guide NSCYC in setting appropriate life goals, clarifying their sense of meaning in life, and seeking interpersonal interactions through constructive and reasonable means to reduce their deviant behavior.

However, there are some limitations to the present study. As a cross-sectional study, it lacked strict attribution for a longitudinal comparison. Moreover, the study relied on a questionnaire survey and lacked alternative methods of investigation. Considering the limitations mentioned above, we should utilize multiple data sources and avoid the singularity of methodology in the future.

## Conclusion

In conclusion, this study showed that perceived social support was negatively related to deviant behavior of NSCYC. After controlling for gender and age, the mediated analyses revealed that interpersonal needs played a mediating role between perceived social support and deviant behavior. The moderated mediation analysis showed that meaning in life moderated the relationship between perceived social support and interpersonal needs, as well as the relationship between perceived social support and deviant behavior. When there is a low sense of meaning in life, perceived social support has a stronger negative impact on interpersonal needs. Conversely, when there is a stronger sense of meaning in life, perceived social support has a negative effect on the deviant behavior of NSCYC.

## Data Availability

The original contributions presented in the study are included in the article/supplementary material, further inquiries can be directed to the corresponding author.
